# Analysis of the prevalence of positivity for tuberculosis infection in formalin-fixed paraffin-embedded tissue

**DOI:** 10.1186/s12879-026-14175-9

**Published:** 2026-08-05

**Authors:** Yu Wu, Sha-sha Hu, Shan-yong Wang, Shu Chen, Bo Wang

**Affiliations:** 1 Department of Pathology, Hainan General Hospital, Affiliated Hainan Hospital of Hainan Medical University, 19 Xiuhua Road, Haikou, Hainan Province 570311 China; 2 Medical Laboratory Center, Hainan General Hospital, Affiliated Hainan Hospital of Hainan Medical University, Hainan Province Haikou, 570311 China

**Keywords:** Pulmonary tuberculosis, Extrapulmonary tuberculosis, Paraffin-embedded tissue, Modified Ziehl-Neelsen acid-fast staining, Fluorescent probe-based polymerase chain reaction

## Abstract

**Background:**

The objective of this study was to analyze the tuberculosis(TB) positivity rate of formalin-fixed paraffin-embedded (FFPE) tissue samples, to explore the difference between the positivity rates of pulmonary tuberculosis (PTB) and extrapulmonary tuberculosis (EPTB), and to compare the value of fluorescent probe-based polymerase chain reaction (PCR) for the detection of *Mycobacterium tuberculosis* DNA (TB-DNA) and modified Ziehl-Neelsen acid-fast staining (Z-N staining) in the diagnosis of tuberculosis.

**Methods:**

A total of 491 patients with histopathological suspected TB admitted to Hainan General Hospital from October 2022 to March 2024 were enrolled. FFPE tissue samples were collected from all participants and examined by fluorescence probe PCR for TB-DNA, acid‑fast staining, and hematoxylin-eosin (H&E) staining. The positive detection rates of TB among different assays were compared. With fluorescent probe PCR results serving as the reference standard, the diagnostic performance of modified Z-N staining was further evaluated. Count data were analyzed using the chi-square test, and a fourfold table was applied to assess the diagnostic efficacy.

**Results:**

TB was detected by PCR in 278 cases and by modified Z-N staining in 89 cases. analysis revealed that age, modified Z-N staining results, musculoskeletal tuberculosis (MSTB), and intra-abdominal tuberculosis were significantly associated with TB-PCR results (*P* < 0.05). The median age was 52 years (interquartile range [IQR]: 37–65 years). Among FFPE tissue samples, the highest proportion of PCR positivity was found in the young group (18–44 years) at 65.97%, while the highest proportion of PCR negativity was seen in the middle-aged group (45–59 years) at 46.84%. The most common sites of EPTB involvement were the pleura, head and neck, and musculoskeletal system, with an overall proportion of 36.46% (179/491). modified Z-N staining was closely correlated with TB-PCR outcomes in both PTB and EPTB subgroups.

**Conclusions:**

The use of PCR detection of TB-DNA is more effective than modified Z-N staining for the diagnosis of PTB and EPTB. It is recommended that FFPE tissue samples are prioritised for screening for *Mycobacterium tuberculosis* by PCR to speed up the diagnosis of TB.

**Clinical trial:**

Not applicable.

## Background

Tuberculosis (TB) represents a significant global public health concern, and the vast majority of TB cases are caused by *Mycobacterium tuberculosis* [1]. The disease primarily accumulates in the lungs but can affect almost all parts of the body, commonly including the respiratory system, the gastrointestinal system, the lymphatic reticulum, the skin, the central nervous system, the musculoskeletal system, the reproductive system, and the liver [2, 3]. In 2024, there were 10.7 million newly notified TB cases worldwide, representing a rise of 0.94% compared with 2022. this epidemiological change indicates the first recovery of global TB response efforts in the post‑pandemic era. An estimated 748,000 new TB cases were reported in China in 2024, accounting for 6.5% of the global incidence. Notably, China, a historically high tuberculosis‑burden country, has been classified into the low-to-moderate tuberculosis prevalence group for the first time, with the national tuberculosis mortality rate dropping to 1.9 per 100,000 population [4]. Nevertheless, global TB prevention and control still face severe challenges. In 2024, global funding for TB prevention, diagnosis and treatment reached only 5.9 billion US dollars, equivalent to one quarter of the funding target set for 2027. This substantial funding shortage highlights an urgent demand for more efficient and accessible diagnostic tools to strengthen TB control strategies world wide. In this context, the present study aimed to evaluate the diagnostic performance of fluorescent probe-based polymerase chain reaction (PCR) for TB detection using formalin-fixed paraffin-embedded (FFPE) tissue samples, to provide evidence for optimizing TB diagnosis in clinical practice.

TB screening and diagnosis primarily rely on multiple modalities, including bacterial culture, clinical manifestation evaluation, immunological testing, pathological examination and radiological imaging. Nevertheless, the diagnosis of extrapulmonary tuberculosis (EPTB) poses substantial clinical challenges. This is attributed to the non-specific clinical and biological features of EPTB, together with the extensive and variable distribution of extrapulmonary lesions. The duration of anti-tuberculosis treatment is mainly determined by the anatomic site of lesions and the severity of disease progression [5]. Extrapulmonary lesions rarely contain viable mycobacteria, and conventional bacteriological tests often produce negative findings. For most suspected EPTB cases, tissue specimens can only be obtained via pathological biopsy. Furthermore, the histopathological manifestations of EPTB frequently manifest as nonspecific inflammatory reactions, which substantially delay definitive diagnosis and timely initiation of treatment. Clinically, it is challenging to collect qualified fresh specimens from EPTB patients for mycobacterial culture. Of note, FFPE tissue specimens used in this study are unsuitable for bacterial culture owing to formalin fixation. Considering these clinical constraints, the combined evidence of typical histopathological changes and positive *Mycobacterium tuberculosis* nucleic acid detection in FFPE tissues is widely accepted as an alternative reference standard for the diagnosis of EPTB. In the absence of culturable *Mycobacterium tuberculosis*, the diagnostic assessment of pathological specimens can be supplemented by modified Ziehl-Neelsen acid-fast staining (Z-N staining) and molecular diagnostics, including microscopic examination for tubercle bacilli and fluorescence probe‑based polymerase chain reaction (PCR). Currently, PCR is recognized as the preferred method for TB detection. This study aimed to compare the *Mycobacterium tuberculosis* detection rates of modified Z-N staining and PCR, and to explore the diagnostic performance differences and clinical application value of the two assays in pulmonary tuberculosis (PTB)and EPTB.

## Methods

### Patient cohorts

A total of 491 consecutive patients pathologically diagnosed with chronic granulomatous inflammation or suspected TB at Hainan General Hospital from October 2022 to March 2024 were retrospectively enrolled in this study. Pathological specimens included puncture biopsy tissues, small biopsy tissues, surgical resection specimens, and pleural fluid samples. All pathological tissue samples were fixed in 10% neutral formalin, dehydrated, embedded in paraffin, and then serially sectioned into 5-µm paraffin sections. Subsequently, FFPE tissue sections were processed for hematoxylin-eosin (H&E) staining, modified Z-N staining, and PCR detection. Inclusion criteria: patients pathologically diagnosed with chronic granulomatous inflammation or suspected TB. Exclusion criteria: malignant tumors.

### PCR

*Mycobacterium tuberculosis* DNA (TB-DNA) was extracted using the AmoyDx FFPE DNA Diagnostic Kit (Amoy Diagnostics Co., Ltd., Xiamen, China) following the manufacturer’s standard operating procedures, as described below. Briefly, each batch of 4–6 paraffin tissue sections was deparaffinized in 1 mL xylene, then rehydrated with 1 mL ethanol. Specimens were air-dried uncapped at 56 °C for 5 min to remove residual ethanol. Next, 180 µL Buffer DTL and 20 µL Proteinase K Solution were added, and tissues were digested at 56 °C for 4 h. Subsequently, 10 µL Buffer DES was added, and the mixture was incubated at 90 °C for 1 h to reverse formalin-mediated DNA crosslinks. Then, 200 µL Buffer DTB and 200 µL absolute ethanol were mixed with the lysate. The mixture was loaded onto a DNA spin column for purification, and purified DNA was eluted for subsequent PCR testing. The reaction system consisted of 12.5µL PCR reaction solution, 3.5µL primer probe mix and 4.0µL purified water (Beijing SinoMD gene diagnostic kit). The target gene sequence tested was IS6110. The reaction program was as follows: The reaction was performed at 37 °C for 2 min, followed by 95 °C for 3 min, 94 °C for 15 s, 60 °C for 35 s and then 45 cycles at 25 °C for 1 min (SLAN-96P). A FAM cycle threshold (Ct) value of less than 37 was considered indicative of a positive result, while a repeat test between 37 and 40 that remained within this range was also considered positive. Conversely, a value greater than 40 was interpreted as a negative result. The positive control for the PCR assay was plasmid containing TB target sequences and internal reference fragments provided in the kit, and the negative control was purified water.

### Modified Ziehl-Neelsen acid-fast staining

The slices were dewaxed in a gasoline-turpentine mixture for two five-minute periods, rinsed under running water for one minute, dyed with drops of phenol alkaline magenta solution for 25 min at room temperature, washed with running water to remove the excess of staining solution, differentiated by 20% sulfuric acid for 30 s, rinsed with running water for 5 min, and the nuclei of the cells were stained lightly with Mayer’s hematoxylin. They were then rinsed with running water for 10 min and dried in an oven at 56 °C. The tissue was rendered transparent with xylene and sealed with neutral gum. Upon microscopic examination, the acid-resistant bacilli exhibited a red coloration, while the nuclei displayed a blue hue.

### H&E staining

The sections were subjected to staining in an automatic dyeing and sealing machine (DAKWE SC2000), and upon microscopic examination, the chromatin in the nucleus and nucleic acids in the cytoplasm were observed to be purplish-blue in color, while components in the cytoplasm and extracellular matrix were seen to be red in color.

### Statistical analysis

SPSS 26.0 software was used for data analysis. Measurement data were presented as **mean ± standard deviation (x ± s)** and compared by the t-test. Count data were expressed as percentages and compared using the chi-square (χ2) test or Fisher’s exact test. A two-tailed *P* < 0.05 was considered statistically significant.

## Results

### Comparison of the diagnostic efficacy of PCR and modified Z-N staining

The analysis results showed that age, modified Z-N staining results, musculoskeletal tuberculosis and intra‑abdominal tuberculosis were significantly correlated with TB-PCR outcomes (*P* < 0.05). Notably, both musculoskeletal and intra-abdominal tuberculosis were significantly related to a higher risk of PCR negativity, whereas no obvious correlation was found between other lesion locations and PCR positive status. The cases included 293 males and 198 females, aged 4 months to 90 years, The proportion of males was 59.67%, The median age was 52 years (interquartile range [IQR]: 37–65 years), The highest proportion of PCR positivity was observed in the young group (18-44years), at 65.97%, while the highest proportion of PCR negativity was observed in the middle-aged group (45–59 years), at 46.84%. Among FFPE tissue samples, the most common sites of EPTB include the pleura, head and neck, as well as the musculoskeletal system, with an overall proportion of 36.46% (179/491). The incidence of musculoskeletal tuberculosis (MSTB) and intra-abdominal tuberculosis was 8.96% and 3.26%, with a positive rate of 40.91% and 43.75% (Table 1).

**Table 1:** Comparison of PCR positivity in different groups of patients with suspected TB

Variables	TB PCR				
	Total (*n* = 491)	Positive (*n* = 278)	negative (*n* = 213)	χ2	*P*
Age, n (%)				17.069	**0.002**
0 ~ 17	22 (4.48)	5 (22.73)	17 (77.27)		
18 ~ 44	144 (29.33)	95 (65.97)	49 (34.03)		
45 ~ 59	158 (32.18)	84 (53.16)	74 (46.84)		
60 ~ 74	139 (28.31)	76 (54.68)	63 (45.32)		
75 ~ 90	28 (5.70)	18 (64.29)	10 (35.71)		
Gender, n (%)				1.740	0.187
Female	198 (40.33)	105 (37.77)	93 (43.66)		
Male	293 (59.67)	173 (62.23)	120 (56.34)		
modified Z-N staining, n (%)				74.882	**< 0.001**
positive	89 (18.13)	87 (31.29)	2 (0.94)		
negative	402 (81.87)	191 (68.71)	211 (99.06)		
Site, n (%)					
Pulmonary	207 (42.16)	124 (44.60)	83 (38.97)	0.210	1.572
NO	284 (57.84)	154 (55.40)	130 (61.03)		
Pleura	73 (14.87)	43 (15.47)	30 (14.08)	0.669	0.182
NO	418 (85.13)	235 (84.53)	183 (85.92)		
Head and neck	62 (12.63)	31 (11.15)	31 (14.55)	1.266	0.261
NO	429 (87.37)	247 (88.85)	182 (85.45)		
Musculoskeletal	44 (8.96)	18 (6.47)	26 (12.21)	4.856	**0.028**
NO	447 (91.04)	260 (93.53)	187 (87.79)		
Lymph node	38 (7.74)	19 (6.83)	19 (8.92)	0.753	0.391
NO	453 (92.26)	259 (93.17)	194 (91.08)		
Axillary	16 (3.26)	11 (3.96)	5 (2.35)	0.991	0.320
NO	475 (96.74)	267 (96.04)	208 (97.65)		
Supraclavicular fossa	15 (3.05)	12 (4.32)	3 (1.41)	3.444	0.063
NO	476 (96.95)	266 (95.68)	210 (98.59)		
Intra-abdominal	16 (3.26)	4 (1.44)	12 (5.63)	6.732	**0.009**
NO	475 (96.74)	274 (98.56)	201 (94.37)		
Reproductive system	10 (2.04)	7 (2.52)	3 (1.41)	0.744	0.388
NO	481 (97.96)	271 (97.48)	210 (98.59)		
Other (pleural fluid, trachea)	10 (2.04)	9 (3.24)	1 (0.47)	0.067	4.631
NO	481 (97.96)	269 (96.76)	212 (99.53)		

The bold values were applied to highlight *P*-values, which had statistically significance (i.e., *P* < 0.05). For each anatomical site variable, “YES” indicates patients with tuberculous lesions at this corresponding site; “NO” indicates all remaining enrolled patients with TB lesions located at other anatomical sites, serving as the pooled reference control group

### Comparison of detection rates of PTB and EPTB

Analysis was performed to explore the correlations of age, gender and modified Z-N staining results with TB-PCR outcomes in PTB and EPTB patients, respectively. In PTB patients, neither age nor gender was significantly associated with TB-PCR positivity (*P* > 0.05). modified Z-N staining status was strongly correlated with TB-PCR results (χ2 = 41.828, *P* < 0.001); all modified Z-N staining-positive cases were TB-PCR positive, and a considerable number of modified Z-N staining-negative samples still presented positive TB-PCR results (Table 2). In EPTB patients, gender showed no significant effect on TB-PCR detection (χ2 = 0.001, *P* = 0.977). By contrast, age was significantly correlated with TB-PCR outcomes (χ2 = 17.04, *P* = 0.002). The 18–44 years age group had the highest TB-PCR positive proportion, while the 0–17 years group exhibited a relatively low detection rate. Similarly, modified Z-N staining was significantly associated with TB-PCR results in EPTB (χ2 = 32.288, *P* < 0.001). Modified Z-N staining-positive specimens showed good consistency with TB-PCR results, and most modified Z-N staining -negative EPTB samples could still be identified as positive by TB-PCR (Table 3). Overall, gender had no influence on TB-PCR detection in either PTB or EPTB populations. Age exerted a significant effect on TB-PCR results only in EPTB patients. Modified Z-N staining was closely correlated with TB-PCR outcomes in both PTB and EPTB subgroups. Compared with modified Z-N staining, TB-PCR demonstrated higher diagnostic sensitivity, especially for EPTB.

**Table 2:** Differences in detection of PTB

Variables	TB PCR				
	Total (*n* = 207)	Positive (*n* = 124)	negative (*n* = 83)	χ2	*P*
PTB					
Age, n (%)				0.972	0.804
0 ~ 17	0 (0.00)	0 (0.00)	0 (0.00)		
18 ~ 44	36 (17.39)	24 (66.67)	12 (33.33)		
45 ~ 59	82 (39.61)	48 (58.54)	34 (41.46)		
60 ~ 74	78 (37.68)	45 (57.69)	33 (42.31)		
75 ~ 90	11 (5.31)	7 (63.64)	4 (36.36)		
Gender, n (%)				3.291	0.700
Female	65 (31.40)	33 (26.61)	32 (38.55)		
Male	142 (68.60)	91 (73.39)	51 (61.45)		
modified Z-N staining, n (%)				41.828	**< 0.001**
positive	48 (23.19)	48 (38.71)	0 (0.00)		
negative	159 (76.81)	76 (61.29)	83 (100.00)		

The bold values were applied to highlight *P*-values, which had statistically significance (i.e., *P* < 0.05)

**Table 3:** Differences in detection of EPTB

Variables	TB PCR				
	Total (*n* = 284)	Positive (*n* = 154)	negative (*n* = 130)	χ2	*P*
EPTB					
Age, n (%)				17.040	**0.002**
0 ~ 17	22 (7.75)	5 (3.25)	17 (13.08)		
18 ~ 44	108 (38.03)	71 (46.10)	37 (28.46)		
45 ~ 59	76 (26.76)	36 (23.38)	40 (30.77)		
60 ~ 74	61 (21.48)	31 (20.13)	30 (23.08)		
75 ~ 90	17 (5.99)	11 (7.14)	6 (4.62)		
Gender, n (%)				0.001	0.977
Female	133 (46.83)	72 (46.75)	61 (46.92)		
Male	151 (53.17)	82 (53.25)	69 (53.08)		
modified Z-N staining, n (%)				32.288	**< 0.001**
positive	41 (14.44)	39 (25.32)	2 (1.54)		
negative	243 (85.56)	115 (74.68)	128 (98.46)		

The bold values were applied to highlight *P*-values, which had statistically significance (i.e., *P* < 0.05)

### Detection of tuberculosis in patients

**Fig. 1:**
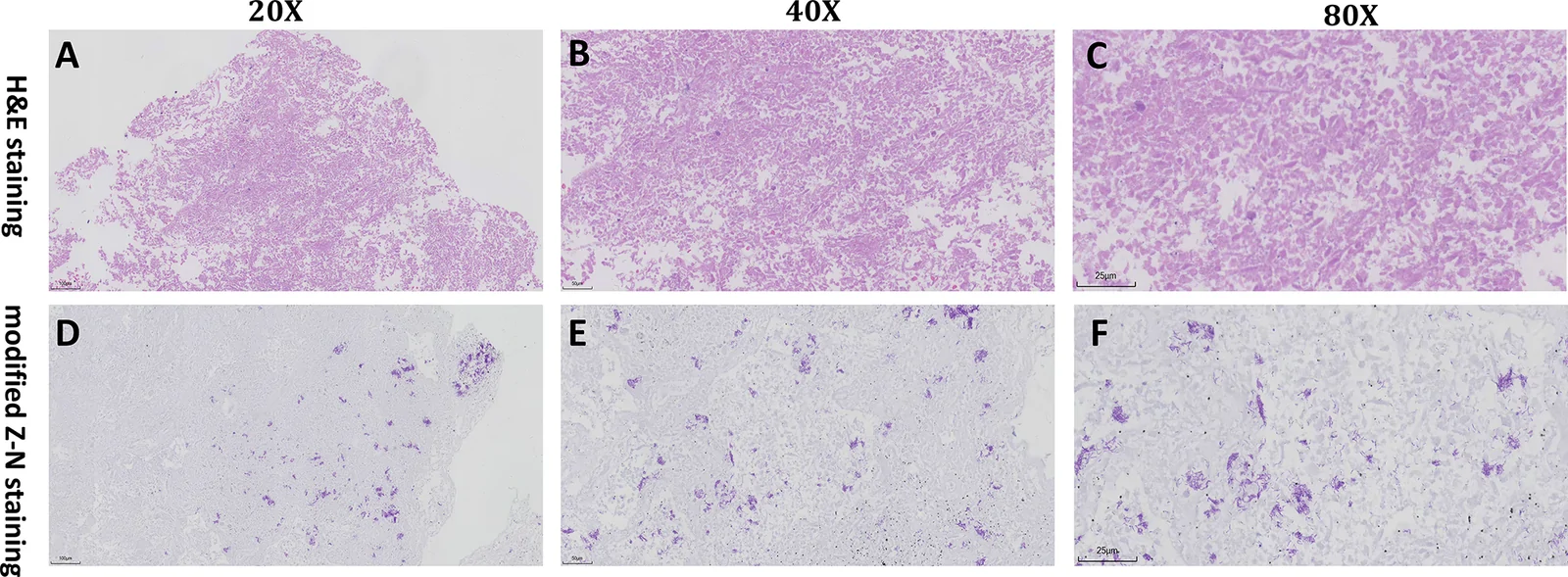
H&E stainingand positive modified Z-N staining. (**A** ~ **C**: H&E staining. **D** ~ **F**: modified Z-N staining)

**Fig. 2:**
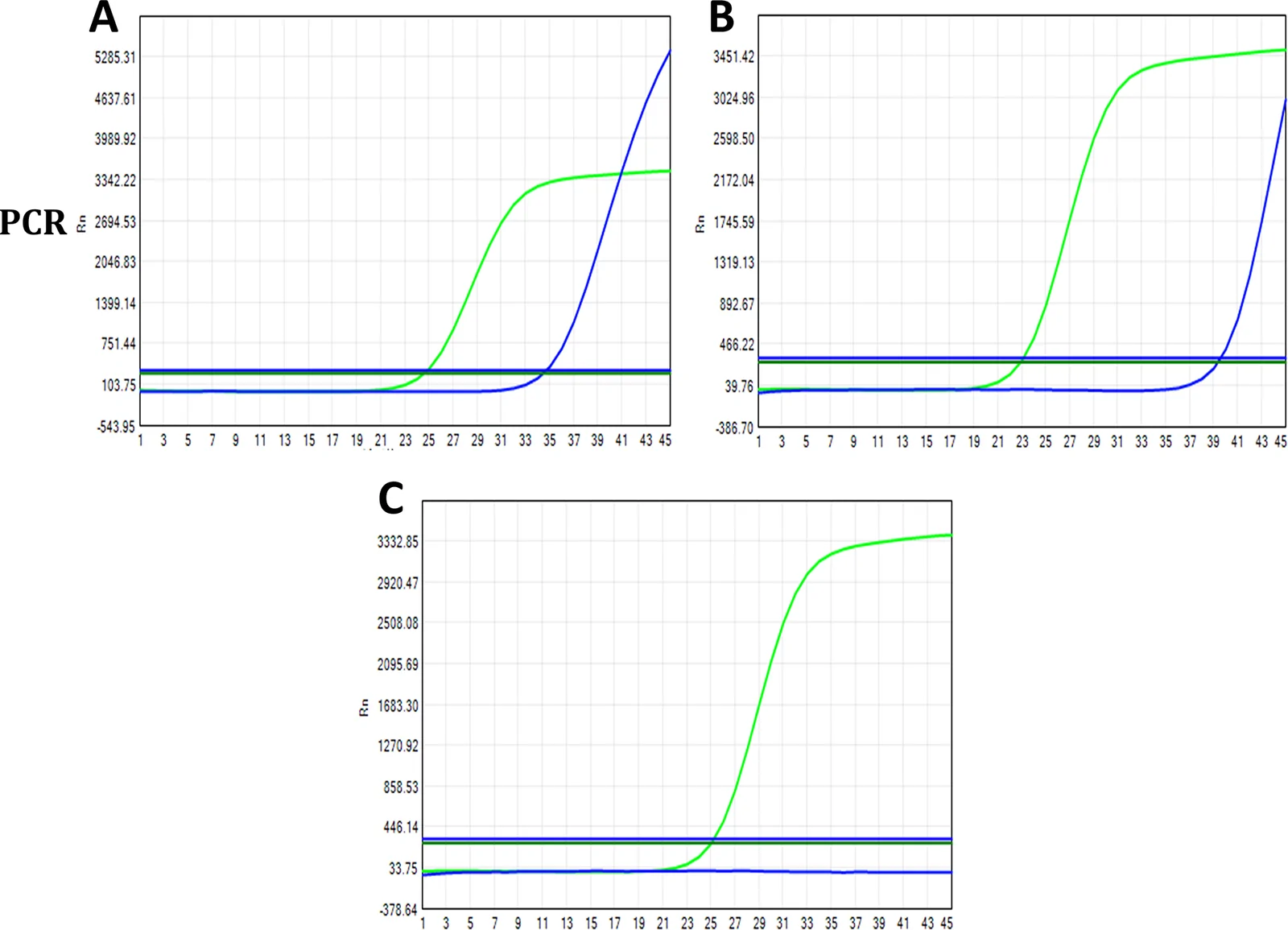
Quantitative fluorescence PCR amplification curves for *Mycobacterium tuberculosis* detection in FFPE tissue specimens. Positive samples presented typical S-type amplification curves, while negative samples showed no amplification signal, supporting Ct-value analysis and validating the performance of the PCR assay. (**A**: Positive PCR curves. **B**: Threshold, need to retest. **C**: Negative PCR curves)

**Fig. 3:**
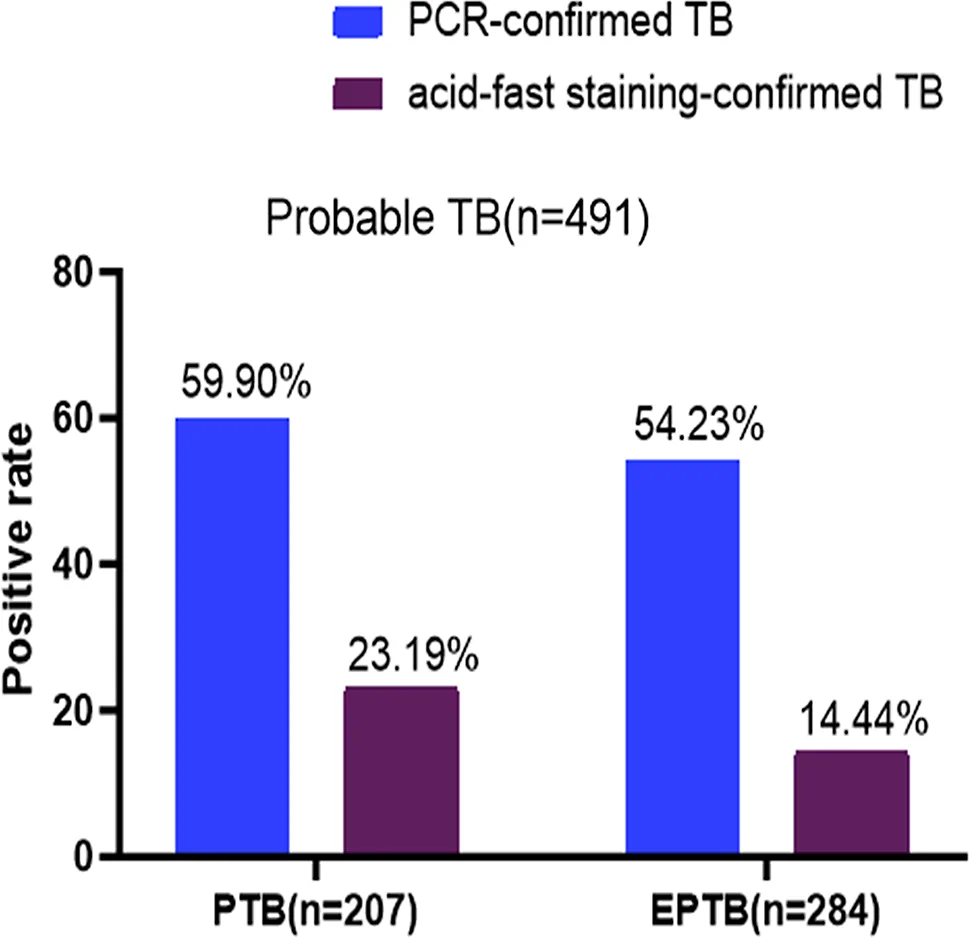
Comparison of PCR and modified Z-N staining detection efficiency in PTB and EPTB

Histopathology revealed necrotizing and granulomatous lesions (Fig. 1A-C) in all 491 patients, positive modified Z-N staining (Fig. 1D-F) in 89 patients, and analyzed the PCR amplification results and corresponding diagnostic parameters among 491 patients (Fig. 2A–C). Among the different histopathologic specimen sites, 207 cases of pulmonary TB had PCR positivity of 59.90% and modified Z-N staining positivity of 23.19%. In 284 cases of EPTB, PCR positivity was 54.23% and modified Z-N staining positivity was 14.44% (Fig. 3).

## Discussion

TB is characterized by the formation of a large necrotizing and granulomatous inflammation, which presents as a rounded collection of macrophages surrounded by lymphocytes. Additionally, peritumoral granulomas with a caseous necrotic center surrounded by epithelioid cells bordered by lymphocytes may be observed. These tuberculous granulomas may also be accompanied by varying amounts of non-necrotizing, neutrophil-rich, mineralized, fibrotic, or cavernous changes [6]. A wide variety of granulomatous diseases exist, including other bacterial and parasitic infections in addition to TB, idiopathic vasculitis, allergies, leukocyte oxidase defects, chemical exposures, and tumors. Furthermore, there is a latent period associated with TB infection, during which the manifestations may be insidious and persist for an extended duration. Consequently, TB may not be the initial factor to be considered in the differential diagnosis, potentially leading to an elusive and delayed diagnosis of TB disease at the pathological level.

This study shows that the majority of PCR-positive samples were from the youth group aged 18–44 years. This may be related to their employment-related migration and high mobility, which increases their risk of infection through contact with contagious patients. Hence, health education should be strengthened for this group to popularize knowledge on TB prevention and treatment. TB poses a considerable global health threat to children. Owing to immature immune systems, infants and young children are particularly vulnerable to progressive and severe tuberculosis manifestations, including disseminated tuberculosis and tuberculous meningitis [7, 8]. Consequently, this group represents a key priority population for tuberculosis vaccination.

In this study, two infants were confirmed positive for *Mycobacterium tuberculosis* infection after vaccination. FFPE tissue specimens were collected from the vaccination site. Microscopic examination revealed typical tuberculous histological features: red-stained unstructured caseous necrosis, focal calcification, peripheral epithelioid cell proliferation, scattered multinucleated giant cells, and variable lymphocyte infiltration around the nodules. The patients presented without chills or fever and were considered likely to have BCG vaccine-associated adverse events (VAEs), which are easily misdiagnosed as TB. For definitive diagnosis and exclusion of VAEs, molecular genetic testing allows rapid and accurate identification of infection caused by *Mycobacterium bovis* BCG [9]. Children with primary or acquired cellular immunodeficiency are more vulnerable to severe or disseminated BCG-related diseases after vaccination [10]. Therefore, screening for a family history of congenital immunodeficiency before BCG vaccination is clinically essential. Given that BCG lesions closely resemble tuberculous pathological changes, accurate differential diagnosis based on a combination of molecular testing and pathological histological diagnosis is therefore particularly critical in clinical practice.

TB is a disease with a high morbidity and mortality rate worldwide. In up to 70–80% of cases, the lungs are the primary organ involved. EPTB represents approximately 15–25% of all TB cases [11]. the incidence of which is increasing, and is thought to be the result of *Mycobacterium tuberculosis* via hematogenous dissemination, lymphatic drainage, or secondary to lesions; it is difficult to make a diagnosis based on imaging features and clinical symptoms, and biopsy is required in many cases [12]. The presence of histopathologic modified Z-N staining and PCR increases the probability of a correct diagnosis of EPTB. Among the paraffin-embedded samples included in this study, the three most common forms of PCR-verified EPTB were Pleural tuberculosis, Head and neck tuberculosis (HNTB), and MSTB, which is consistent with the findings reported in the literature [13]. Among these, MSTB causes significant morbidity due to late onset of symptoms and lack of accurate diagnosis, and among musculoskeletal involvement, spinal tuberculosis (also known as Pott’s disease) is the most prevalent manifestation, which may result in severe neurological complications. Early diagnosis and intervention help reduce the risk of spinal deformity and optimise clinical outcomes [14].

The present study revealed a prevalence of 57.84% for EPTB, with a PCR positivity rate of 54.23%. This proportion is relatively higher than those described in previous studies. The discrepancy may be attributed to our exclusive use of histopathological specimens. With the widespread application of minimally invasive sampling techniques, pathological biopsy has become increasingly accessible for accurate etiological diagnosis. PTB can be readily diagnosed based on typical clinical manifestations and routine pathogenetic examinations. In comparison, the diagnosis of EPTB remains challenging due to low bacterial load in extrapulmonary lesions, insufficient sensitivity of conventional laboratory tests, and frequent morphological overlap with inflammatory or neoplastic lesions, which often necessitates biopsy and comprehensive pathological evaluation. Existing studies have demonstrated that EPTB patients have poorer treatment and long-term prognosis than PTB patients, and HIV co-infection, advanced age and central nervous system tuberculosis further increase their mortality risk. Although PTB is associated with higher overall long-term mortality, high-risk EPTB subgroups still present unfavorable outcomes and require close clinical monitoring and intervention [15, 16]. Consistent with substantial prior clinical evidence, nucleic acid PCR assays achieve superior diagnostic sensitivity for *Mycobacterium tuberculosis* compared with conventional Ziehl-Neelsen acid-fast staining [17]. PCR enables identification of trace amounts of mycobacterial DNA, whereas acid-fast staining only detects specimens with high mycobacterial loads [18]. Nevertheless, most published studies focus solely on sputum samples and lack systematic histopathological validation using FFPE extrapulmonary tissues, which limits their translational value for clinical practice. Caseous necrosis, granulomatous lesions, and the presence of acid-fast bacilli on Ziehl-Neelsen staining are robust morphological features highly suggestive of mycobacterial infection; however, these histological features alone cannot confirm *Mycobacterium tuberculosis* infection. Non-tuberculous mycobacteria are also capable of triggering granulomatous lesions that yield positive acid-fast staining results. Therefore, molecular PCR targeting *M. tuberculosis*-specific genes is necessary to establish a definitive diagnosis of EPTB. In the present study, we cross-compared histopathological, PCR, and modified Z-N staining results from histopathologically confirmed positive cases. Histopathological examination identified characteristic granulomatous lesions even in FFPE specimens with low mycobacterial load that tested negative via modified Z-N staining, while PCR demonstrated better pathogen-specific detection performance than routine modified Z-N staining. The three diagnostic modalities possess complementary advantages: Ziehl-Neelsen smear microscopy enables rapid, low-cost preliminary screening, histopathology provides intuitive morphological evidence of tissue lesions, and PCR delivers definitive molecular identification of the causative pathogen. Combined utilization of these three approaches substantially improves the overall diagnostic accuracy of EPTB using FFPE tissue blocks. Consistent with the statistical data, nearly all Z-N-positive specimens generated positive PCR signals, while the PCR positive rate dropped dramatically in Z-N-negative tissues. This discrepancy accounts for the weak PCR amplification observed in a subset of EPTB samples. Lesions with sparse bacilli easily lose trace mycobacterial DNA during dewaxing and nucleic acid purification, and formalin-induced genomic DNA fragmentation further compromises amplification efficiency. Collectively, heterogeneous mycobacterial loads across biopsy lesions serve as the primary factor driving inconsistent PCR detectability among FFPE EPTB specimens. At present, molecular PCR assays are widely used for rapid detection of *Mycobacterium tuberculosis* in sputum, blood and puncture aspirates, owing to simple operating procedures and short turnaround times [19–22]. Prior to the widespread adoption of molecular testing, acid-fast staining acted as a routine auxiliary technique for pathological tuberculosis diagnosis [23]. However, its inherently low sensitivity frequently generates false-negative results. In contrast, PCR demonstrates high species specificity, making it better suited for detecting tuberculous lesions in diverse extrapulmonary pathological specimens and facilitating timely, accurate auxiliary diagnosis of EPTB [24].

Several limitations of this study should be acknowledged. First, this was a single center, retrospective cross sectional investigation. All specimens were collected from clinical pathological samples, and unavoidable selection bias may limit the generalizability of our findings. Second, PCR cannot distinguish between active viable tuberculosis infection and residual dead mycobacterial DNA from previous infection, which may potentially cause false-positive results.

## Conclusions

In conclusion, PCR demonstrates superior diagnostic performance and discriminatory capability compared with modified Z-N staining for tuberculosis detection. Therefore, we recommend routine PCR testing for all pathologically suspected tuberculous specimens, regardless of the presence or absence of necrotic tissue. Combined application of PCR and modified Z-N staining can effectively improve the overall detection sensitivity, and is strongly recommended to optimize the pathological diagnosis of tuberculosis.

## Data Availability

All data generated or analysed during this study are included in this published article. Further inquiries can be directed to the corresponding author upon reasonable request.

## Abbreviations

PCR: Fluorescent probe-based polymerase chain reaction
PTB: Pulmonary tuberculosis
EPTB: Extrapulmonary tuberculosis
MSTB: Musculoskeletal tuberculosis
